# A Modified Femtosecond Laser Technique for Anterior Capsule Contraction Syndrome

**DOI:** 10.1155/2020/9423267

**Published:** 2020-12-24

**Authors:** Marco Marenco, Pietro Mangiantini, Luca Scuderi, Alessandro Lambiase, Marta Sacchetti

**Affiliations:** Department of Sense Organs, University Sapienza of Rome, Rome, Italy

## Abstract

Anterior capsule contraction syndrome (ACCS) is a rare, late complication of cataract surgery, associated with impairment of visual function. In this paper, we describe a new surgical technique to treat ACCS by femtosecond laser procedure. The femtosecond laser was used to perform an anterior capsulotomy with a customized size, in order to avoid IOL damage. After ophthalmic viscosurgical device injection in the anterior chamber, the anterior capsule flap was separated from the IOL surface by gentle hydrodissection. This manoeuvre enabled an easy and safe removal of the fibrotic material by vitreal microscissors. Our technique allowed a complete removal of the fibrotic material and opening of the capsule, with immediate complete visual acuity recovery without IOL damage. In conclusion, femtosecond laser appears to be safe and effective for treatment of ACCS with long-lasting efficacy.

## 1. Introduction

Anterior capsular phimosis or anterior capsule contraction syndrome (ACCS) is one of the late complications following cataract surgery [1–3].

ACCS is characterized by an excessive fibrosis response after capsulorhexis, which causes reduction in the size of the anterior capsulotomy and capsular bag diameter. It is often associated with impairment of visual function [3–8]. Rarely, this zonular traction may lead to IOL dislocation and retinal detachment [1, 9].

Once ACCS occurs, removal of fibrous membrane may be required to improve visual function. Currently, the most common technique for treating ACCS is Nd:YAG laser anterior capsulotomy [10]. However, most severe cases often require surgical removal of the fibrous membrane [11–13]. Recently, the use of femtosecond laser technology to treat ACCS has been proposed with different techniques [10, 11, 14].

We describe a novel technique which consists of femtosecond laser capsulotomy followed by surgical removal of the fibrotic material, in a patient with severe ACCS, performed one year after cataract surgery.

## 2. Materials and Methods

### 2.1. Surgical Procedure

After corneal topical anesthesia, lid speculum is applied, and the patient is placed under the femtosecond laser system LenSX® (Alcon, Fort Worth, TX, USA) in order to maximize suction applanation. The procedure starts with eye docking and centering. Once the operator acquires all information from LenSX® anterior OCT and establishes cutting parameters, capsulotomy may be performed. Specifically, live OCT imaging is used to establish the depth of capsulotomy by taking into consideration the shape and thickness of the anterior phimosis. The diameter was set in correspondence with the higher gap between the posterior surface of the capsule and the anterior surface of the IOL (see video in Supplementary Materials). The capsulotomy width performed was based on the patient's maximal mydriasis.

At the end of the femtosecond laser procedure, the surgical bed is rotated under the operating microscope, the previous paracenteses of cataract surgery are manually reopened, and the ophthalmic viscosurgical device (OVD) is inserted into the anterior chamber. Gentle hydrodissection is performed to release the adherences between the anterior capsule and the anterior surface of the IOL. The removal of the anterior capsule, previously incised with the femtosecond laser, is easily performed with rhexis forceps with a continuous curvilinear capsulorhexis-like movement.

Residual fibrotic tissue bridges are cut by 23-gauge vitreal microscissors through the femtosecond laser previous incision. OVD is then removed from the anterior chamber, and the procedure is completed with stromal hydration of the surgical wounds.

## 3. Results

**Figure 1:**
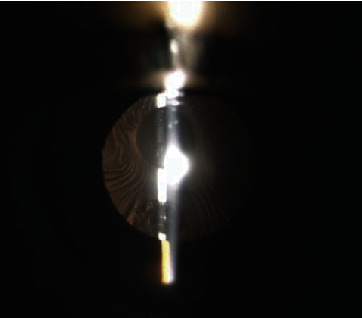
Slit lamp exam showing the presence of thick anterior capsule phimosis, involving visual axis.

LenSx® femtosecond laser system was used to treat ACCS in a 74-year-old woman who came to our observation complaining diminished visual acuity in her left eye. She reported a clinical history of myopia and referred phacoemulsification cataract surgery with implantation of ARTIS PL E (Cristalens, France) in her left eye (LE) 11 months before. She denied any known medical condition and history of ocular inflammation or trauma. Ophthalmological examination revealed a best-corrected visual acuity of (BCVA) of 20/40 in the LE and 20/20 in the fellow eye. Slit lamp examination showed the presence of anterior capsule fibrosis involving the visual axis in the LE (Figure 1).

**Figure 2:**
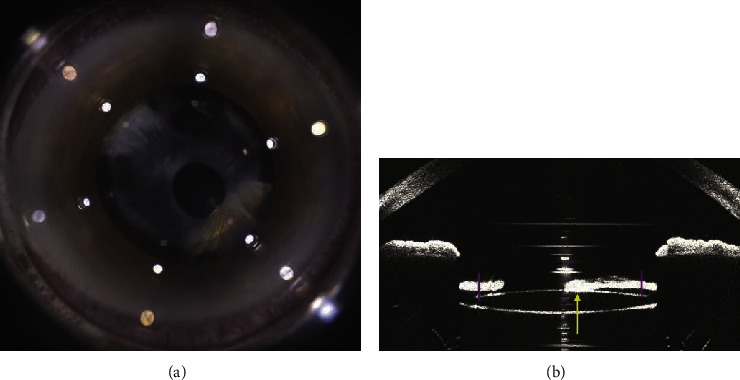
Femtosecond laser system (a) and LenSX® anterior optical coherence tomography (OCT) (b) allow the visualization of fibrotic adherence between anterior capsule and the anterior surface of the IOL .

Intraocular pressure was 13 mmHg in both eyes, and ophthalmoscopic fundus examination was normal. LenSX optical coherence tomography (OCT) of the anterior segment showed a complete adherence of the anterior capsule to the anterior surface of the IOL, with different phimosis thickness ranging from 50 to 531 *μ*m (Figure 2).

**Figure 3:**
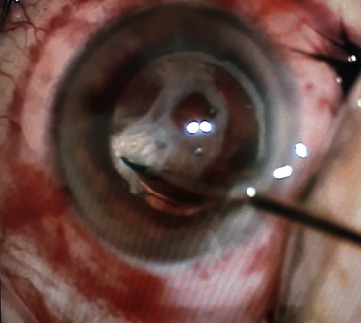
Intraoperative image of residual fibrotic tissue removal using 23-gauge vitreal microscissors following femtosecond laser capsulotomy.

Given the thickness and irregularity of the anterior capsule fibrosis and the adherence between the anterior capsule and the IOL, the LenSx® femtosecond laser system was used to treat ACCS with the procedure described. In this patient, the incision depth for the capsulotomy was set at 600 *μ*m (manually selecting a capsule delta up of 280 *μ*m and capsule delta down of 320 *μ*m), in order to include the entire fibrotic capsule thickness; given the highly fibrotic material, the laser pulse energy was increased to 6 *μ*J (from the standard 4 *μ*J used in cataract surgery), and the spacing was decreased to 5 *μ*m horizontally and 3 *μ*m vertically. The capsulotomy width (5 mm) was based on the patient's maximal mydriasis. Residual fibrotic tissue bridges after the femtosecond incision were cut by 23-gauge vitreal microscissors (Alcon-Grieshaber, Fribourg, Switzerland) (Figure 3).

**Figure 4:**
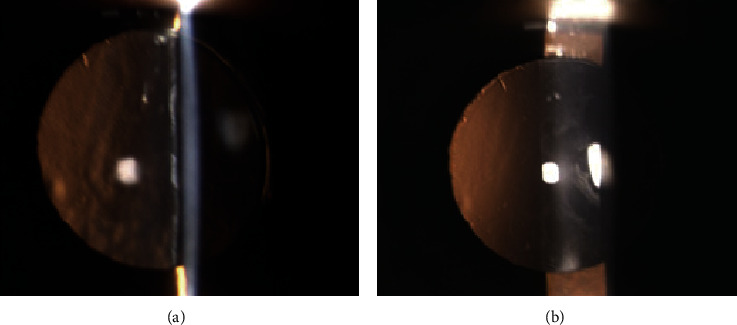
The day after the procedure, slit lamp examination shows complete removal of the fibrotic phimosis and absence of intraocular inflammation (a). After one-year follow-up, anterior capsule opening was unchanged (b).

The day after the procedure, the patient's BCVA recovered to 20/20 in her LE; slit lamp examination showed no inflammation in the anterior chamber, complete removal of capsular fibrosis, and IOL stability. Intraocular pressure was 12 mmHg (Figure 4(a)). The patient was treated with betamethasone 0.2% and chloramphenicol 0.5% (Betabioptal® eye drops, Thea Farma, France) 1 drop 4 times a day for 7 days. After one year of uneventful follow-up, the patient had a BCVA of 20/20; slit-lamp examination showed no recurrence of anterior capsule fibrosis and IOL stability (Figure 4(b)).

## 4. Discussion

ACCS is a rare late complication of cataract surgery, with onset during the first week following surgery and progressing for several months [15]. Small diameter capsulorhexis, pseudoexfoliation syndrome, chronic ocular inflammation, high myopia, and advanced age have been associated with ACCS. Nd:YAG laser anterior capsulotomy is the most commonly used technique for ACCS treatment [10]. Despite the high success rate, this technique has unpredictable efficacy in restoring visual acuity in patients with thick fibrosis. Moreover, several complications have been described such as IOL pitting, cystoid macular edema, or residual fibrotic material in the anterior chamber that may cause inflammation and secondary glaucoma [1, 11, 16–19].

Alternatively, surgical removal of the fibrous membrane may be performed by capsulorhexis to peel the fibrotic capsule, using microincisional scissors or vitrector-cut capsulotomy [16, 20, 21]. This procedure allows a better centration of the IOL, but may be complicated by lens dislocation in case of zonular weakness. Zinkernagel et al. described a minimally invasive surgical technique using standard 23-gauge vitreoretinal forceps and horizontal scissors to bimanually remove the fibrotic anterior capsule and to perform haptic amputation [16]. However, a potential risk of IOL or iris damage may be associated with the use of vitrector scissors.

Recently, the use of femtosecond laser technology has been proposed to treat severe ACCS [10]. In fact, femtosecond laser allows to extend the capsulorhexis more precisely and less traumatically than cutting by Nd:YAG laser, ensuring a 360 degree overlap of the IOL optic. In addition, the incision depth of capsulotomy may be located between the IOL and the anterior capsule surface, allowing a complete removal of the fibrotic tissue with low risk of IOL damage [11].

Specifically, femtosecond laser can cut hard tissues such as highly fibrotic and adherent anterior capsules, allowing treatment of severe ACCS cases [10]. Ibarz et al. described a case of bilateral simultaneous femtosecond laser-assisted capsulotomy for the treatment of severe anterior capsule contraction that had caused complete occlusion of the anterior capsule in one eye and partial occlusion in the fellow eye one month after cataract surgery. The authors favored the femtosecond laser in order to minimize the risk of IOL folding, inflammation, and intraocular pressure peaks, and the procedure resulted safe and easy to perform despite the extreme case of anterior capsule contraction. The femtosecond laser capsulotomy parameters were similar to the ones set in our technique. Capsulotomy was completed with forceps and scissors in one eye and a Sinskey hook in the fellow eye. After one-month follow-up, the patient presented 20/20 best-corrected visual acuity, and the IOL showed no signs of damage [22].

In this report, we describe the use of femtosecond laser to treat ACCS in the presence of a very thick and irregular capsule fibrosis and its efficacy after one year of follow-up. We propose the use of femtosecond laser capsulotomy followed by OVD injection in the anterior chamber and surgical removal of the cut fibrotic capsule using rhexis forceps and vitreal microscissors. Unlike previously reported techniques, we did not use OVD before femtosecond laser treatment because OCT images clearly showed the presence of a gap between the capsule and the IOL.

In conclusion, we describe an innovative and safe treatment for ACCS using femtosecond laser technology combined with bimanual surgical procedure to remove very thick and extended ACCS, allowing a prompt and long-lasting visual acuity recovery that remained stable after one year of follow-up. The high costs of femtosecond laser and the lack of standardized protocols due to anecdotal reports represent the main limitation to a routinely use of this technique. In addition, the high costs of the technique represent an issue in developing countries. Hopefully, femtosecond laser-assisted procedures can become more accessible in the short term, especially for patients who present with extensive capsular fibrosis and adherences. Further studies with large number of patients will help in the future to establish standardized laser settings and procedures.

## Data Availability

The data used to support the findings of this study are available from the corresponding author upon request.

## Supplementary Materials

Supplementary Materials Video: video shows the femtosecond laser capsulotomy of anterior capsule phimosis. The procedure starts with eye docking and centering with LenSX®. The operator establishes cutting parameters by LenSX® optical coherence tomography. After establishing a pupil diameter of 5.4 mm, the operator sets the femtosecond laser capsulotomy depth and performs femtosecond laser anterior capsulotomy.
